# Retrospective analysis of hemophilia B in Turkey: identifying main characteristics and treatment options

**DOI:** 10.1016/j.rpth.2024.102588

**Published:** 2024-10-10

**Authors:** Bülent Zülfikar, Başak Koç, Fahri Şahin, Hatice İlgen Şaşmaz, Kaan Kavaklı, Can Balkan, Ali Bülent Antmen, Sinan Akbayram, Birol Güvenç, Vahap Okan, Emine Türkkan, Canan Albayrak, Davut Albayrak, Nazan Sarper, Tülin Tiraje Celkan, Orhan Ayyıldız, Salih Aksu, Türkan Patıroğlu, Zafer Şalcıoğlu, Adalet Meral Güneş, Yasemin Altuner Torun, Ümran Çalışkan, Hüseyin Tokgöz, Yılmaz Ay, Gül Nihal Özdemir, Mehmet Sönmez, Ekrem Ünal, Ahmet Fayik Öner, Nil Güler, Osman Alphan Küpesiz, Hale Ören, Serap Karaman, Ayşegül Ünüvar, Mehmet Dağlı, Ahmet Muzaffer Demir, Murat Söker, Bülent Alioğlu, Zühre Kaya, Aylin Canbolat Ayhan, Zafer Bıçakçı, Yusuf Ziya Aral, Muhlis Cem Ar

**Affiliations:** 1 Hereditary Bleeding Disorders Unit, Istanbul University Oncology Institute, Istanbul, Turkey; 2 Department of Internal Diseases, Division of Hematology, Ege University Medical Faculty Hospital, Izmir, Turkey; 3 Department of Pediatric Hematology, Cukurova University Faculty of Medicine, Adana, Turkey; 4 Department of Children’s Health and Diseases, Division of Pediatric Hematology, Ege University Medical Faculty Hospital, Izmir, Turkey; 5 Department of Pediatric Hematology and Oncology, Acibadem Hospital, Adana, Turkey; 6 Department of Pediatric Hematology and Oncology, Gaziantep University Medical Faculty, Gaziantep, Turkey; 7 Department of Internal Diseases, Division of Hematology, Cukurova University Faculty of Medicine, Adana, Turkey; 8 Department of Internal Diseases, Division of Hematology, Gaziantep University Medical Faculty, Gaziantep, Turkey; 9 Department of Pediatric Hematology-Oncology, Ministry of Health Prof. Dr. Cemil Taşçıoğlu City Hospital, Istanbul, Turkey; 10 Department of Pediatric Hematology and Oncology, Ondokuz Mayıs University Faculty of Medicine, Samsun, Turkey; 11 Department of Pediatric Hematology-Oncology, Samsun Medical Park Hospital, Samsun, Turkey; 12 Department of Pediatric Hematology, Kocaeli University School of Medicine, Kocaeli, Turkey; 13 Department of Pediatric Hematology and Oncology, İstinye University, Istanbul, Turkey; 14 Department of Internal Medicine, Division of Hematology, Dicle University Medical Faculty, Diyarbakır, Turkey; 15 Department of Internal Medicine, Division of Hematology, Hacettepe University Faculty of Medicine, Ankara, Turkey; 16 Division of Pediatric Immunology and Oncology, Losante Children’s and Adult Hospital, Ankara, Turkey; 17 Department of Pediatric Hematology, Kanuni Sultan Süleyman Training and Research Hospital, Istanbul, Turkey; 18 Department of Pediatric Hematology and Oncology, Uludağ University Faculty of Medicine, Bursa, Turkey; 19 Department of Pediatric Hematology, Necmettin Erbakan University Meram Medical Faculty Hospital, Konya, Turkey; 20 Department of Pediatric Hematology, Pamukkale University School of Medicine, Denizli, Turkey; 21 Department of Internal Medicine, Division of Hematology, Karadeniz Technical University Faculty of Medicine, Trabzon, Turkey; 22 Department of Pediatrics, Division of Pediatric Hematology and Oncology, Erciyes University Faculty of Medicine, Kayseri, Turkey; 23 Department of Pediatric Hematology and Oncology, Yuzuncu Yil University Faculty of Medicine, Van, Turkey; 24 Department of Internal Medicine, Division of Hematology, Pamukkale University School of Medicine, Denizli, Turkey; 25 Department of Pediatric Hematology, Akdeniz University Faculty of Medicine, Antalya, Turkey; 26 Department of Pediatrics, Division of Pediatric Hematology, Dokuz Eylul University Faculty of Medicine, İzmir, Turkey; 27 Department of Pediatrics, Division of Pediatric Hematology-Oncology, Istanbul University Istanbul Faculty of Medicine, Istanbul, Turkey; 28 Department of Internal Medicine, Division of Hematology, Selcuk University Faculty of Medicine, Konya, Turkey; 29 Department of Internal Medicine, Division of Hematology, Trakya University Faculty of Medicine, Edirne, Turkey; 30 Department of Pediatric Hematology, Dicle University Medical Faculty, Diyarbakır, Turkey; 31 Department of Pediatric Hematology and Oncology, Ankara Training and Research Hospital, Ankara, Turkey; 32 Department of Pediatric Hematology, Gazi University Faculty of Medicine, Ankara, Turkey; 33 Department of Pediatric Oncology and Hematology, Istanbul Medeniyet University Faculty of Medicine, Istanbul, Turkey; 34 Department of Pediatric Hematology and Oncology, Atatürk University, Erzurum, Turkey; 35 Department of Pediatric Hematology, Adnan Menderes University School of Medicine, Aydın, Turkey; 36 Department of Internal Medicine, Division of Hematology, Istanbul University – Cerrahpasa, Cerrahpasa Faculty of Medicine, Istanbul, Turkey

**Keywords:** factor IX, hemophilia B, joint diseases, prophylaxis, retrospective studies, surgery

## Abstract

**Background:**

Hemophilia B (HB), an X-linked recessive inherited bleeding disorder, exhibits a high prevalence among males.

**Objectives:**

To present the first national cohort of persons with HB to define the demographics, clinical characteristics, and treatment patterns in Turkey.

**Methods:**

This multicenter, retrospective study included 433 alive persons with HB registered in 35 centers between 1961 and 2018. Analyses were performed by age subgroups (0-17 years, 18-64 years, and ≥65 years), disease severity by factor levels (severe, <1 IU/dL; moderate, 1-5 IU/dL; mild, >5 IU/dL). Additionally, patients were stratified based on the initiation year of follow-up at the relevant study center, creating 2 periods: 1993-2006 (referred to as period A) and 2007-2018 (referred to as period B).

**Results:**

Predominantly male (98.6%), the median age at data entry was 22.1 years (*n* = 429). The majority (49.0%) had moderate HB, followed by severe (30.0%) and mild (15.7%) disease. Of the 377 patients with complete treatment details, 209 (55.4%) were under prophylaxis from their diagnosis onwards, while 79 patients (21.0%) only received on-demand treatment. Additionally, 89 patients (23.6%) initially underwent on-demand treatment and later were switched to prophylaxis. Knees were the primary site of bleeding and the most frequently intervened joints. Most of the major (47.5%) and minor (53.3%) orthopedic procedures were carried out in persons with severe HB, while half of radioactive synovectomy procedures were performed on persons with moderate HB.

**Conclusion:**

This paper describes the demographics, clinical characteristics, and treatments patterns of a large cohort of alive persons with HB on a national scale.

## Essentials

• Hemophilia B, a factor IX deficiency, causes extended bleeding after injuries or surgery.
• Past information noting disease and treatment details was collected on persons with hemophilia B in Turkey.
• Sixty percent had severely low factor levels, and the majority (70%) were on prophylactic treatment.
• Knees bled most, often treated; surgeries included radiosynovectomy, dental, and circumcision.

## 1 Introduction

Hemophilia B (HB), an X-linked, inherited bleeding disorder, constitutes nearly 15% to 20% of the overall hemophilia population [1]. A recent meta-analysis in 2019 presented a new global estimate of persons with hemophilia, indicating an approximately 5-fold increase compared with the previous year [2,3]. The same meta-analysis reported an estimated prevalence of 3.8 cases and a birth prevalence of 5.0 cases per 100,000 males for HB [2]. According to a 2020 global survey conducted by the World Federation of Hemophilia, participating countries reported 33,076 persons with HB, indicating that the total number of persons with HB accounted for one-fifth of the total number of persons with hemophilia A [4].

The severity of HB has been defined by factor (F)IX levels in the plasma: <1 IU/dL are classified as severe, 1 to 5 IU/dL as moderate, and 5 to 40 IU/dL as mild deficiency [1]. The severe form is characterized by a high frequency and prolonged duration of spontaneous bleeding episodes, primarily occurring in joints (hemarthrosis), especially in elbows, knees, ankles, muscles, and soft tissues. With time, recurrent chronic synovitis and hemophilic arthropathy result in progressive joint and cartilage destruction, stiffness, severely limited movement, and chronic pain which inevitably necessitates radiosynovectomy or orthopedic intervention [5,6]. Successful management of persons with HB relies on the prevention and/or treatment of bleeding episodes with replacement of deficient FIX through the use of either plasma-derived or recombinant therapies [7]. In recent years, the introduction of new extended half-life FIX products has resulted in improved prophylaxis outcomes and a significant increase in treatment compliance [8]. Factor concentrates were not available in Turkey before 1993. Between 1993 and 2007, factor replacement therapy was accessible through a special program and for on-demand treatment (ODT) only. From 2007 onward, prophylaxis using factor concentrates of 4500 units/wk became widely available following full reimbursement of factor products for all persons with hemophilia with a factor activity level ≤1% and/ or with ≥3 bleeds/mo, regardless of prior treatment status. While almost all previously untreated patients were put on prophylaxis following the reimbursement, the switch rate in previously treated patients was lower than expected. Based on the practical experiences of physicians and experts in the field, the switch initially involved adolescents and young adults who were eager to learn self-infusion, while patients at advanced ages were reluctant to switch from ODT to prophylaxis. Extended half-life products did not enter the market until 2024. Inhibitors continue to be the most clinically significant challenge in replacement treatment, with a reported occurrence in 1% to 3% of persons with HB, although a recent paper has revealed inhibitors in 9.1% of persons with severe HB [9,10]. Anaphylaxis and proteinuria are other important complications associated with the development of inhibitors in HB [11].

According to the latest available data (2014), there are a total of 5738 persons with hemophilia in a population of 81 million living in Turkey [12]. Persons with hemophilia A and HB were reported to be 4860 and 878, respectively. Although the observed prevalence rate appears to be lower than global figures, it is essential to note that no new data has been released since 2014, and there has not been a nationwide comprehensive registry or study on persons with HB. The objective of this large-scale registry study was to gather retrospective data on the demographics, clinical characteristics, and treatment patterns of living persons with HB in Turkey.

## 2 Methods

### 2.1 Participants and design

This national, multicenter, retrospective study aimed to include all living persons with HB who were confirmed to be alive at study enrolment period and seen and treated between 1961 and 2018 at 35 treatment centers in Turkey. All available patient data, extracted from medical records and patient files between March 20, 2018, and January 31, 2019, were uploaded into an electronic data collection system using electronic data collection forms. Ethical approval was obtained from the Clinical Research Ethics Committee of Istanbul University (Istanbul, Turkey) on January 12, 2018 (approval number: 01). Informed consent was waived due to the retrospective nature of the study.

### 2.2 Data collection and definitions

Patient demographic characteristics included age at the date of data entry, sex, date of birth, family history, age at the date of switch to prophylaxis, and admission date to the center. Furthermore, throughout the patient’s follow-up period at the relevant study center, various additional information on FIX levels (the lowest detected factor level in the medical records), inhibitor development and titers, joint status (“joint involvement at first examination” was defined as total number of affected joints at first examination), treatment-related adverse events (such as anaphylaxis and presence of proteinuria), orthopedic interventions (categorized as major, minor, and radioactive synovectomy [RS]), nonorthopedic interventions, viral tests (hepatitis B surface antigen [HBsAg], hepatitis B surface antibody [anti-HBs], hepatitis C virus antibody [anti-HCV], anti-HIV, and HCV-RNA/hepatitis B virus DNA when appropriate), intracranial hemorrhage (ICH), and the clinical presentation of patients recorded at the last visit (including neurologic sequela and musculoskeletal deformities) were collected. Musculoskeletal deformities were accepted as deformities resulting from hemophilia, while neurologic sequelae were accepted as sequelae following ICH. Low-titer inhibitors were defined as <5 BU/mL, while high-titer inhibitors were defined as ≥5 BU/mL [1].

Patients were categorized into 3 groups based on their treatment type during the follow-up period: those who received prophylaxis only (PX group), those who received ODT only (ODT group), and those who initially received ODT and later switched to prophylaxis (ST group). Differences based on age groups (0-17 years, 18-64 years, and ≥65 years) and the severity of disease: severe (FIX, <1 IU/dL), moderate (FIX, 1-5 IU/dL), or mild (FIX, >5 IU/dL) were also analyzed. Given that factor concentrates were reimbursed in 2007 in Turkey, this time point was selected to define groups for analysis of different treatment outcomes: period A (1993-2006), when a limited number of factor concentrates were available through a named patient access program; and period B (2007-2018), when factor replacement therapy became widely available following reimbursement.

### 2.3 Statistical analysis

All quantitative variables collected in the study were summarized using the following parameters: sample size (*n*), mean, SD, median, and IQR when applicable. Qualitative variables were presented as numbers and percentages. Within-treatment group comparisons were performed using Kruskal–Wallis test, considering the nonnormal distribution indicated by the Shapiro–Wilk test, and in cases of significance, the Mann–Whitney U-test was used for paired group comparisons. The statistical analysis was performed using the SPSS statistical software program (SPSS 25.0, SPSS Inc). The level of significance was considered as *P* < .05.

## 3 Results

### 3.1 Study population and demographics

Patient data were retrospectively collected between March 2018 and February 2019, comprising 551 living persons with HB from 35 hemophilia centers (both pediatric and adult) in Turkey. Patients whose admission dates to the centers preceded 1993 were excluded due to the unavailability of factor concentrates in Turkey during that period. Consequently, the final analysis included a total of 433 patients. The demographic and clinical characteristics are given in Table 1. Most patients (67.0%) started follow-up in period B. Male patients constituted 98.6% of the cohort. The median (IQR) age at data entry was 22.1 (11.6-32.8) years (*n* = 429), with the majority (59.6%) being adults. Positive family history was prevalent in 44.6% of the patients, while it was unknown for 33.9% of patients. Moderate HB was observed in 49.0% of patients, followed by severe (30.0%) and mild (15.7%) HB. The factor levels were unknown in 23 patients (5.3%). Among the cohort, 19 patients were positive for clotting factor inhibitors, with 12 (2.8%) having low titer and 7 (1.6%) having high titer.

**Table 1:** Baseline demographics and disease characteristics.

Variables	Values
Beginning year of follow-up,^a^*n* (%)	
1993-2006 (period A)	129 (29.8)
2007-2018 (period B)	290 (67.0)
Unknown	14 (3.2)
Sex, *n* (%)	
Male	427 (98.6)
Female	6 (1.4)
Age, mean ± SD	
0-17 y (*n* = 173)	9.7 ± 4.8
18-64 y (*n* = 246)	32.6 ± 11.6
≥65 y (*n* = 10)	73.6 ± 8.0
Family history, *n* (%)	
Yes	193 (44.6)
No	93 (21.5)
Unknown	147 (33.9)
Disease severity (factor IX level), *n* (%)	
Severe (<1 IU/dL)	130 (30.0)
Moderate (1-5 IU/dL)	212 (49.0)
Mild (>5 IU/dL)	68 (15.7)
Unknown	23 (5.3)
Inhibitor titer, *n* (%)	
LT <5 BU/mL	12 (2.8)
HT ≥5 BU/mL	7 (1.6)
No inhibitor detected	382 (88.2)
Unknown	32 (7.4)

HT, high titer; LT, low titer.

a Beginning year of follow-up of the patients at the reporting center was divided into 2 periods based on the accessibility to factor replacement therapy in Turkey.

Fifty-six patients had incomplete or missing treatment records. Of the 377 patients with complete treatment details, 209 patients (55.4%) were on PX from the initiation of follow-up at the study center, and 79 patients (21.0%) received ODT only. Eighty-nine out of 377 patients (23.6%) initially received ODT but were switched to prophylaxis (ST group). Regarding the switch dates of patients in the ST group, the median age was 13.1 years, with a range from 4 months to 51.9 years (*n* = 87). Among all 3 treatment groups, persons with moderate HB were the most frequent: 48.3% of patients in the PX group (101/209) and 46.1% in the ST group (41/89) had moderate HB. Additionally, the rate of severe HB was also comparable with that of moderate HB: in the PX group, 37.3% (78/209) had severe HB, while 42.7% (38/89) of the ST group were persons with severe HB. Although 53.2% of patients in the ODT group (42/79) had moderate HB, mild HB also constituted a high proportion (31/79 patients, 39.2% of ODT). Regardless of the disease severity, the PX rate among patients aged over 18 years (138/256 patients, 53.9%) was higher compared with those under 18 years (69/173 patients, 39.9%). Regardless of age, the majority of the persons with severe HB (78/121 patients, 64.5%) received PX. Among persons with moderate HB, 54.9% (101/184 patients) were on PX, while those with mild HB had higher rates of ODT (31/60 patients, 51.7%). Treatment regimens associated with the severity of HB and age are outlined in Table 2.

**Table 2:** Disease severity and age subgroups of persons with hemophilia B according to the treatment regimens.

Disease severity^a^ and age subgroup, *n* (%)	PX	ODT	ST	Total
Severe hemophilia B				
0-17 y	26 (56.5)	3 (6.5)	17 (37.0)	46 (100.0)
18-64 y	49 (68.1)	2 (2.7)	21 (29.2)	72 (100.0)
≥65 y	2 (100.0)	-	-	2 (100.0)
Age unknown	1 (100.0)	-	-	1 (100.0)
Subtotal	78 (64.5)	5 (4.1)	38 (31.4)	121 (100.0)
Moderate hemophilia B				
0-17 y	33 (51.6)	15 (23.4)	16 (25.0)	64 (100.0)
18-64 y	66 (57.4)	24 (20.9)	25 (21.7)	115 (100.0)
≥65 y	1 (25.0)	3 (75.0)	-	4 (100.0)
Age unknown	1 (100.0)	-	-	1 (100.0)
Subtotal	101 (54.9)	42 (22.8)	41 (22.3)	184 (100.0)
Mild hemophilia B				
0-17 y	7 (28.0)	15 (60.0)	3 (12.0)	25 (100.0)
18-64 y	14 (45.1)	15 (48.4)	2 (6.5)	31 (100.0)
≥65 y	3 (75.0)	1 (25.0)	-	4 (100.0)
Age unknown	-	-	-	0
Subtotal	24 (40.0)	31 (51.7)	5 (8.3)	60 (100.0)
Total, *N*	203	78	84	365

ODT, on-demand treatment; PX, prophylactic treatment; ST, switch from on-demand treatment to prophylaxis.

a Disease severity was not known for 6 in PX group, 1 in ODT group, and 5 in ST group.

### 3.2 Joint involvement at first examination

Data on joint involvement at first examination were available for 303 patients. Overall, patients had a median of zero joint involvement (range, 0-9) at the first examination (*n* = 303). Regardless of the disease severity or treatment type, the mean number of involved joints differed among 3 age subgroups (*P* < .001). Subsequently, paired comparisons of age subgroups revealed that in the 18 to 64 years subgroup, it was 3 times higher than in those in the 0 to 17 years subgroup (1.21 vs 0.43; *P* < .001). Persons with severe HB had the highest mean number of joint involvements, followed by persons with moderate and mild HB in decreasing order, however, the difference was not statistically significant (1.11 vs 0.81 vs 0.54, respectively; *P* = .16). On the other hand, the mean number of affected joints was significantly different among the age subgroups based on treatment types (Table 3). Paired comparisons of age subgroups revealed that the patients in the 18 to 64 years subgroup had higher joint involvement at the first examination in all 3 treatment groups compared with the 0 to 17 years subgroup (*P* < .001, *P* = .002, and *P* = .01 in PX, ODT, and ST groups, respectively).

**Table 3:** Joint involvement at first examination according to disease severity, age, and treatment regimens.

Treatment regimens	Age subgroups				Disease severity subgroups			
	0-17 y	18-64 y	≥65 y	*P* value^a^	Severe hemophilia B	Moderate hemophilia B	Mild hemophilia B	*P* value^a^
PX								
N	59	83	2	<.001	50	73	17	.59
Mean (SD)	0.51 (0.84)	1.53 (1.58)	0.50 (0.71)		1.32 (1.85)	1.04 (1.16)	0.71 (0.85)	
Median (IQR)	0 (0-1)	1 (1-2)	0.50 (0-NA)		1 (0-2)	1 (0-2)	0 (0-1.50)	
Min to max	0-3	0-9	0-1		0-9	0-4	0-2	
ODT								
N	26	36	4	.009	5	35	25	.29
Mean (SD)	0.12 (0.43)	0.64 (0.90)	0.25 (0.50)		0.00 (0.00)	0.51 (0.89)	0.36 (0.64)	
Median (IQR)	0 (0-0)	0 (0-1)	0 (0-0.75)		0 (0-0)	0 (0-1)	0 (0-1)	
Min to max	0-2	0-4	0-1		0-0	0-4	0-2	
ST								
N	35	40	0	.01	31	35	5	.80
Mean (SD)	0.57 (0.88)	1.23 (1.25)	-		1.06 (1.32)	0.80 (1.05)	0.80 (0.84)	
Median (IQR)	0 (0-1)	1 (0-2)	-		1 (0-2)	0 (0-1)	1 (0-1.50)	
Min to max	0-4	0-5	-		0-5	0-4	0-2	

max, maximum; Min, minimum; NA, not available; ODT, on-demand treatment; PX, prophylactic treatment; ST, switch from on-demand treatment to prophylaxis.

a Kruskal–Wallis test was conducted to compare all 3 age and disease severity subgroups within each treatment group, respectively. In case of a significance, subsequent paired comparisons of age subgroups were presented in the text.

### 3.3 Surgery

According to the medical records, a total of 142 orthopedic interventions were identified. The numbers of orthopedic and other surgical interventions are presented in Table 4. Among the orthopedic interventions, 40 (28.2%), involving 31 patients, were classified as major procedures, while 15 interventions (10.5%) affecting 11 patients were categorized as minor procedures. Additionally, a total of 87 RSs were performed, accounting for 61.3% of the orthopedic interventions, and involving 48 patients. Among these 48 patients, 21 underwent multiple RS procedures. Fifteen patients required a second course of RS in the same joint, performed within 1 to 9 years. Furthermore, 3 patients had a third RS procedure for the same knee joint (years unknown). The majority of the orthopedic interventions (63.4%) were conducted in the PX group, whereas 25.4% were in the ST group. Persons with severe HB underwent 47.5% of major interventions and 53.3% of minor interventions. Additionally, 41.4% of RS interventions were carried out in persons with severe HB, while half of RS interventions (50.6%) were performed on persons with moderate HB. Focusing on the anatomical distribution, half (50%) of the major surgeries, 45% of the minor surgeries, and 63.2% of RS procedures were performed on knee joints.

**Table 4:** Number of orthopedic and other interventions on follow-up period according to treatment regimens, year, and type of intervention.

Year of the intervention^a^	Orthopedic interventions			Other interventions	Total
	Major surgery	Minor surgery	RS		
PX					
1993-2006	6	2	11	25	44
2007-2018	19	4	39	93	155
Year unknown	0	0	9	21	30
Subtotal	25	6	59	139	229
ODT					
1993-2006	0	0	0	11	11
2007-2018	4	1	3	35	43
Year unknown	3	0	3	8	14
Subtotal	7	1	6	54	68
ST					
1993-2006	2	3	11	15	31
2007-2018	4	5	11	36	56
Year unknown	0	0	0	4	4
Subtotal	6	8	22	55	91
Patients with no treatment details	2	0	0	6	8
Total	40	15	87	254	396

ODT, on-demand treatment; PX, prophylactic treatment; RS, radiosynovectomy; ST, switch from on-demand treatment to prophylaxis.

a Year of intervention was grouped into 2 periods based on the accessibility to factor replacement therapy in Turkey: 1993-2006, a limited number of factor concentrates were available through a named patient program; 2007-2018, factor replacement therapy became widely available following reimbursement.

The 254 nonorthopedic interventions included various procedures, with dental procedures (78 procedures, 30.7%) and circumcision (114 procedures, 44.9%) being the predominant types. The remaining 62 procedures included general surgery operations, such as inguinal hernia operation, appendectomy, or pilonidal cyst operation, as well as urological operations like prostatectomy, and dermatologic procedures, such as nail and nevus interventions. Out of the 23 patients with unknown factor levels, 2 patients in the 18 to 64 years age subgroup underwent 4 RSs, which were related to knee and elbow joints equally. Additionally, 4 patients had a total of 4 other interventions, 3 of which were circumcisions.

### 3.4 Viral disease status

In our study, the results of hepatitis testing were also documented. Out of the 286 patients who were tested for HBsAg, only 11 individuals (3.9%) had a positive test result. Among this subset, 6 patients were negative for anti-HBs. In the group of 283 patients who were tested for anti-HBs, 80 patients (28.3%) displayed positivity for this marker. Notably, among those, the majority (74 patients) had negative HBsAg. Regarding hepatitis C testing, among the 287 patients with a test result, 30 patients (10.5%) exhibited positive results for anti-HCV, and only 3 of them demonstrated positivity for HCV-RNA. There was no HIV positivity recorded among 277 tested patients.

### 3.5 ICH

In our cohort, a total of 14 cases of ICH were recorded; of which only 8 were attributed to trauma, while the causes of remaining cases were unknown. Eight patients (57.1%) presented with moderate HB, while 5 persons had severe HB. Notably, with the exception of 2 patients (factor levels were 4% and 15%), all ICH cases had factor levels below 2 IU/dL. The majority of the patients (*n* = 13) were under the age of 18 years, including 1 under 12 months of age at the time of ICH, with a median age of 8 years. The remaining patient was 25 years old. Additionally, 2 out of 14 patients were receiving ODT, while the remaining 12 patients were equally distributed between PX and ST groups.

### 3.6 Safety and clinical presentation at last visit

The incidence of anaphylactic reactions to infusions was minimal (*n* = 3) and proteinuria was observed in only 4 patients. During the last visit, 79 out of 390 patients with available information (20.3%) had developed musculoskeletal deformities. Among this group, 36 patients (45.6%) had moderate HB, while severe HB was present in 32 patients (40.5%). The majority of these patients (57.0%) were undergoing PX. Lastly, only 7 (*n* = 400, 1.8%) patients had neurologic sequelae.

## 4 Discussion

Hemophilia is characterized by bleeding into joints, muscles, and internal organs which can be life-threatening, and it is closely related to arthropathy resulting from recurrent bleeding episodes. In this national retrospective cohort study, we report the demographic and clinical characteristics of alive persons with HB in Turkey. Almost 40% of patients were in the pediatric age group at data entry. The mean age was 23.1 years (median: 22.1 years), lower than in similar studies in other countries [13,14]. As factor concentrates became available in Turkey starting from 1993 and received reimbursement in 2007, it may be of note that some patients before 2007 were lost to follow-up, possibly moving abroad to get access to factor replacement or succumbing at an early age to the fatal effects of the disease. This could potentially account for the lower number of elderly patients in our study population compared with other countries.

Almost one-third of the patients (30.0%) had severe HB, with nearly 40% of these being under the age of 18 years. The ratio of disease severity (moderate/severe) in our study was 1.6, higher than in certain countries, such as Sweden (0.7), Spain (0.7), United Kingdom (0.5), and Germany (0.4), but similar to France (1.4) and Italy (1.3) [13]. It is important to emphasize that among the 212 persons with moderate HB in our study, the factor levels of 129 (61%) fell within the range of 1 to 2 IU. These individuals may exhibit a clinical course similar to severe HB and could have been diagnosed more frequently. Consequently, we believe that our study findings hold significance, particularly in raising awareness among our colleagues for persons with moderate HB in our country. Another study also reported a similar ratio of moderate disease, with the rate of mild HB stated as 27.9% [14]. Mild disease rates are less commonly reported in the literature. In a publication by the World Federation of Hemophilia, mild hemophilia was estimated at 18% to 34% [15]. Similarly, another study reported this rate as 39% [16]. In our study, 15.7% had mild HB, possibly due to underdiagnosis or fewer medical records due to their less frequent seeking of medical help.

Approximately half of our cohort had a family history; however, data on family history were lacking in one-third of the cases. Therefore, our results should be interpreted with caution, as Kasper and Lin [17] reported positive family history in 57% and 70% of persons with severe and mild-moderate HB, respectively. The inhibitor rate in our cohort was 4.4%, which is nearly 2 to 3 times higher than the rates observed in persons with HB [9]. These rates may vary based on the severity of the disease; a recent study found that 9.1% of the persons with severe HB had inhibitors [10], while in our study, half of the inhibitors were in persons with severe HB. Nevertheless, only 1.6% of cases had high-titer inhibitors requiring treatment. The higher inhibitor rate in our cohort might have been influenced by the inclusion of patients with low titer, as the latter have been associated with an increased frequency of false positivity, mainly due to the varying assessment methods over time.

Initiating prophylaxis with factor replacement at an early age is considered the gold standard of care in hemophilia and is known to be superior to ODT [1,18]. In our study, irrespective of age and disease severity, the proportion of patients receiving prophylaxis was approximately 3 times higher than those undergoing ODT. These findings are similar to a study by Oldenburg et al. [19] (88.9% and 11.1%; respectively) but differ from the global data in the 2018 World Bleeding Disorders Registry report where ODT was 2.5 times more frequent than PX [20]. Our study findings revealed that PX was the preferred treatment option for both adult and younger patients. Moreover, persons with severe HB were predominantly managed with prophylaxis as recommended [21], while ODT was more commonly preferred for persons with mild HB. PX was also the primary choice for persons with moderate HB in our study. This observation can be interpreted in light of the factor levels of those patients, with 61% having levels between 1 and 2 IU/dL.

The most prevalent sites of bleeding were joints and muscles, with the knees being the most affected, as documented in previous reports [1,22,23]. In our study, persons with severe HB exhibited the highest number of involved joints at the initial examination, closely followed by those with moderate HB, due to the prevalence of relatively low factor levels in our cohort of moderate HB. Additionally, adult patients demonstrated 3 times more involved joints than those under 18 years, irrespective of treatment type and disease severity. This difference was consistent across all 3 treatment subsets examined. Five patients with severe disease receiving ODT displayed no joint involvement, possibly due to milder clinical manifestations taking into account their factor levels.

Hemophilic arthropathy, the primary cause of musculoskeletal deformities, may severely limit mobility, and if left untreated, lead to joint and cartilage destruction requiring surgical intervention [6,24]. When primary interventions such as physical therapy, medical treatments, and conservative measures fail to alleviate pain and improve mobility in the early stages, surgical interventions become a viable consideration [24]. In our cohort, half of the major and minor orthopedic interventions were performed in patients with severe disease, with this trend increasing over time. However, the majority of other interventions, primarily including dental and circumcision procedures, were conducted in persons with moderate HB. Interestingly, contrary to earlier reports, RS which was the majority of the orthopedic interventions, were in persons with moderate HB [25,26]. The knee was the most frequently treated joint, a correlation that aligns well with its high frequency of involvement observed at initial examination.

Despite new safer treatment options, hemophilic patients still remain at risk for hepatitis viruses and HIV. In Turkey, a national immunization program for hepatitis B was introduced in 1998 [27]. However, the seropositivity of hepatitis B antibody was observed in only 6 patients within our cohort. It was reassuring to note that HBsAg was also detected in a limited number of patients. Similarly, the positivity for HCV-RNA was also relatively low. According to Ministry of Health, HIV incidence among hemophilic patients remains low in Turkey [28]. This study also did not identify any instances of HIV positivity.

Even though ICH is a rare complication, it is the most dangerous bleeding event occurring in persons with hemophilia [29]. ICH events were recorded in 14 patients, who mainly had moderate diseases, yet factor levels of the majority of patients (87.5%) were below 2 IU/dL. A similar ratio was also observed in patients aged <18 years of age. Seven (1.8%) patients had neurologic sequelae. ICH-associated mortality rates could not be given because of the nature of the study.

This study has several limitations. First, while interpreting the outcomes it should be kept in mind that the study includes only data from alive persons with HB. Additionally, information on the race or ethnicity of patients was not collected. Nevertheless, Turkey is a country with a rich tapestry of ethnicities. It is important to note that, given the evolving political landscape in the past decades, the study population may be diverse, encompassing individuals from Turkey and Middle Eastern countries. Second of all, due to the retrospective nature of the study, there were missing data, including factor levels, which might have biased some of our results. Additionally, reaching data of the patients in the ODT group was challenging, potentially leading to the underrepresentation of these patients in the cohort. Lastly, diagnosis of some mild and moderate cases might have been missed since those patients may not frequently present to clinical attention, impacting the distribution of mild, moderate, and severe cases in the cohort.

## 5 Conclusion

There was limited information on the clinical presentation and management of persons with HB in the Turkish population. This study represents the largest cohort of alive persons with HB in Turkey with data on disease severity, joint disease, and treatment choices. The study population mainly consisted of persons with moderate HB. Prophylaxis was the predominantly preferred type of management. Prophylaxis rates decreased in the older population (aged 65 years and older). Given the paucity of information on characteristics and management of persons with HB, the outcomes of this study could shed light on the current situation of HB in Turkey and provide healthcare providers and health authorities with valuable data. However, these results should be justified and challenged with those of a prospective large-scale registry.
